# Giant sellar aneurysm presenting with arginine vasopressin deficiency: a rare case report

**DOI:** 10.3389/fendo.2026.1737157

**Published:** 2026-06-03

**Authors:** Xiaolong Zhang, Pengfei Wang, Baogen Pan

**Affiliations:** 1 Hebei North University, Zhangjiakou, China; 2 Neurosurgery Department of Hebei General Hospital, Shijiazhuang, Hebei, China

**Keywords:** diabetesinsipidus, hypopuitarism, pituitary adenoma, sellar region aneurysm, sellar region

## Abstract

Giant aneurysms are rare vascular anomalies that can induce symptoms through compression of neural structures. This report describes a case of a giant sellar aneurysm initially manifesting as diabetes insipidus (DI), challenging conventional diagnostic paradigms for sellar mass lesions. Although DI typically arises from antidiuretic hormone (ADH) deficiency, its presentation as the primary symptom of a giant sellar aneurysm is exceedingly uncommon. The distinctiveness of this case lies in the occurrence of DI, likely due to aneurysmal compression of the pituitary gland, underscoring the imperative to include aneurysms in the differential diagnosis of sellar lesions. Giant sellar aneurysms are frequently misdiagnosed as pituitary adenomas, particularly when atypical symptoms are present. Through multidisciplinary collaboration, this case was successfully managed, yielding valuable clinical insights. This report not only advances the understanding of giant sellar aneurysms but also provides a foundation for future research to mitigate misdiagnosis and enhance patient quality of life.

## Introduction

Giant aneurysms, defined as those exceeding 25 mm in diameter, are uncommon vascular lesions that may compress neural structures, leading to symptoms such as headache, visual disturbances, and hypopituitarism (1). Sellar aneurysms account for approximately 1%–2% of all intracranial aneurysms, with those associated with pituitary dysfunction being particularly rare (2). Diabetes insipidus, characterized by polyuria and polydipsia and typically caused by ADH deficiency, is a well-established clinical entity. However, its presentation as the initial symptom of a giant sellar aneurysm has not been previously documented.

The uniqueness of this case is highlighted by the patient’s initial presentation with DI, ultimately diagnosed as a giant sellar aneurysm. This finding challenges traditional clinical perceptions of sellar masses. The successful multidisciplinary management of this case offers valuable experience for the diagnosis and treatment of analogous cases in the future.

## Case presentation

A 60-year-old woman presented with dizziness, blurred vision for 8 days, and increased urine output of approximately 6 L per day. Her medical history included hypertensive intracerebral hemorrhage 22 years prior, treated with burr hole surgery, with full recovery and no significant sequelae. She had a 22-year history of hypertension, with a peak blood pressure of 200/110 mmHg, controlled pharmacologically.

On admission, the patient was alert, oriented, and cooperative. Bilateral visual acuity was impaired. Pupils measured approximately 3.0 mm with prompt light reflexes. Cardiopulmonary and abdominal examinations were unremarkable. Muscle strength was normal in all extremities, without involuntary movements. Reflexes were symmetric and within normal limits. No neck stiffness or abnormal neurological signs were detected.

**Figure 1:**
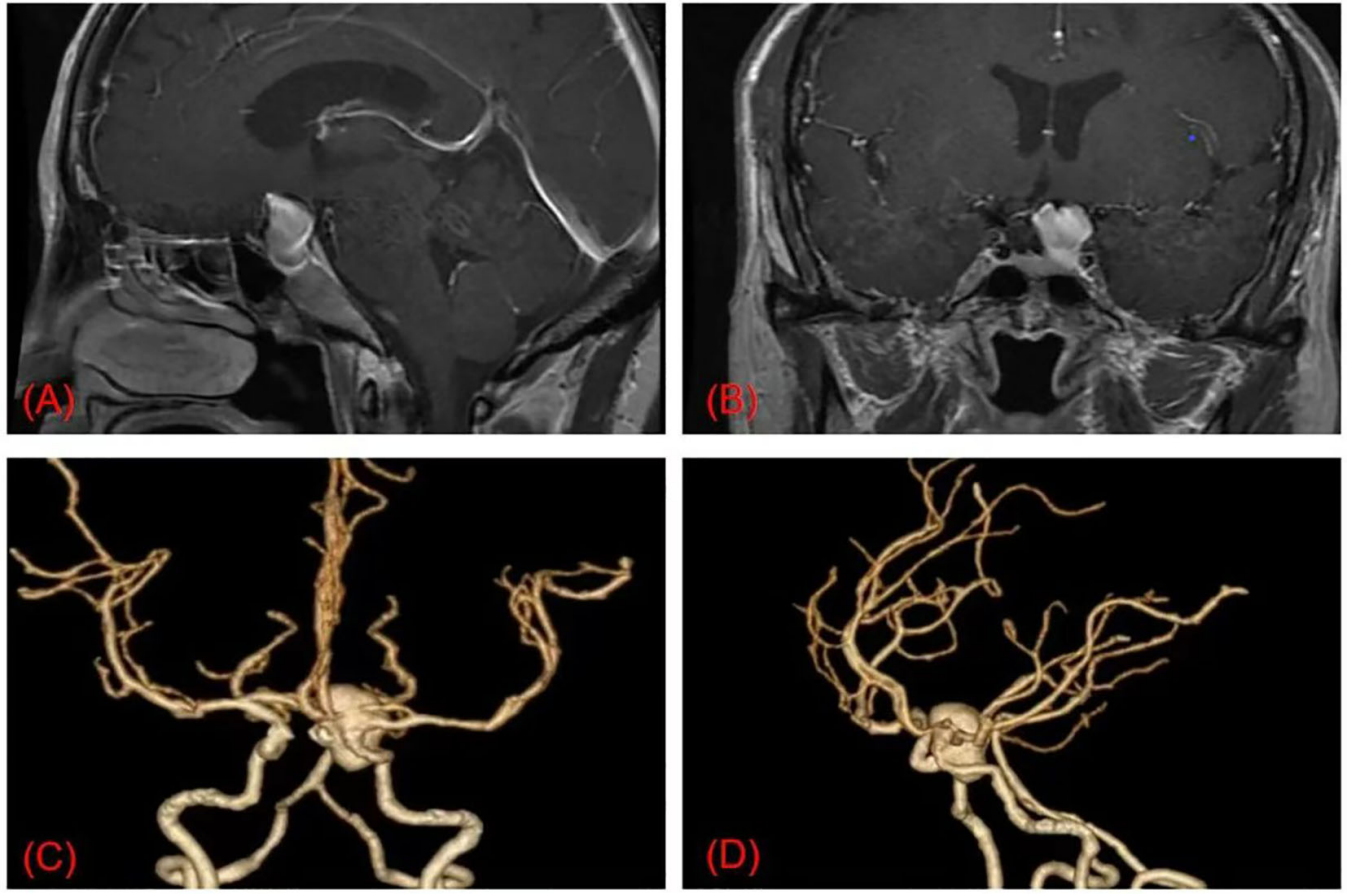
**(A, B)** T2-weighted magnetic resonance images of the pituitary gland, demonstrating an abnormally enhancing signal within the sellar region. This lesion is immediately adjacent to the left internal carotid artery, with no discernible plane of cleavage between them on imaging. **(C, D)** Cranial computed tomographic angiography (CTA) established the diagnosis of an aneurysm arising from the communicating segment of the left internal carotid artery, with the dome projecting into the sella turcica.

Non-contrast head computed tomography (CT) revealed a left frontal bone defect and left frontal lobe encephalomalacia. A mass lesion was noted in the left cavernous sinus. Pituitary MRI (MRI) demonstrated an abnormal enhancing signal in the sellar region, closely associated with the left internal carotid artery (**Figures 1A, B**). Head CTA (CTA) confirmed an aneurysm at the communicating segment of the left internal carotid artery, projecting into the sella turcica (**Figures 1C, D**). Laboratory tests showed serum osmolality of 340 mmol/L, Serum Sodium of 145 mmol/L, urine osmolality of 254 mmol/L, and urine specific gravity of 1.002. Prolactin levels were mildly elevated. Endocrine evaluation revealed laboratory evidence of panhypopituitarism. Specifically, the early morning serum cortisol was significantly low at 2 μg/dL, accompanied by a suppressed adrenocorticotropic hormone (ACTH) level of 8 pg/mL. Additionally, the free thyroxine (FT4) level was 0.7 ng/dL, which is below the reference range.

**Figure 2:**
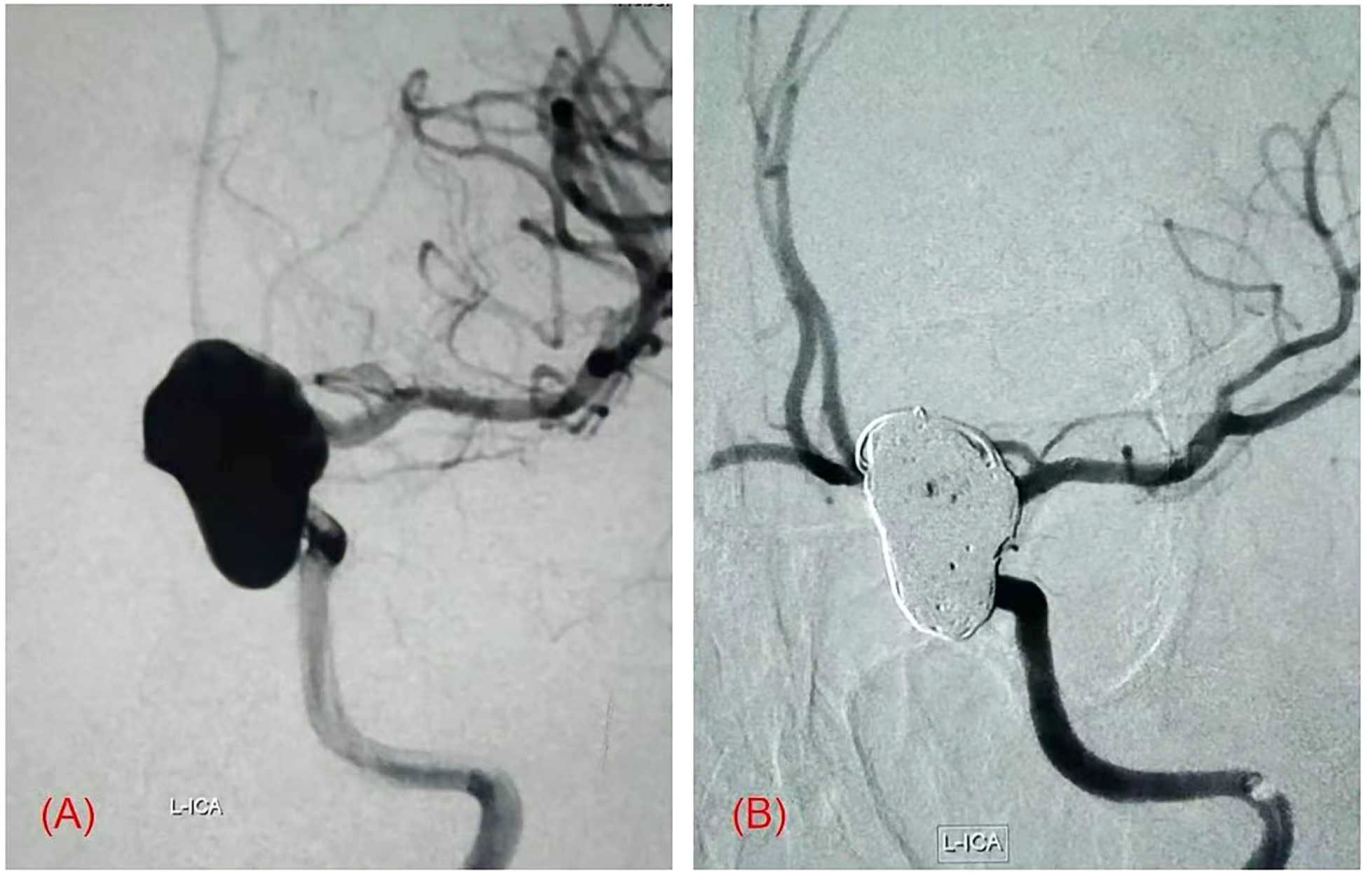
**(A)** Cerebral angiography demonstrating an aneurysm arising from the communicating segment of the left internal carotid artery. **(B)** Follow-up angiography showing satisfactory occlusion of the aneurysm with preservation of parent artery patency.

Based on clinical and radiological findings. the patient’s dizziness, visual impairment and DI were attributed to compression of the pituitary stalk, posterior pituitary, and optic chiasm by the giant aneurysm. The patient received 1 mg of intravenous desmopressin, resulting in a significant reduction in urine output. Daily intravenous desmopressin was continued. After comprehensive discussion with the family, cerebral angiography was performed, confirming a left internal carotid artery communicating segment aneurysm (**Figure 2A**). The patient subsequently underwent placement of a flow-diverting stent and coil embolization of the aneurysm with 8 coils. Postoperative angiography demonstrated successful aneurysm occlusion with parent artery patency (**Figure 2B**). Hormone replacement therapy was continued postoperatively. The patient’s visual acuity and DI symptoms improved markedly. She was discharged in stable condition. At the 6-month follow-up, while on oral desmopressin 0.1 mg three times daily, her visual fields and DI had significantly improved, with urine output maintained within the normal range. Oral administration of levothyroxine sodium 25 μg once daily and hydrocortisone 10 mg once daily.The patient remains under follow-up for monitoring of endocrine hormone levels and related symptoms.

## Discussion

The differential diagnosis of sellar region masses encompasses a broad spectrum of pathologies, including pituitary adenomas, cysts, craniopharyngiomas, meningiomas, germ cell tumors, inflammatory or infiltrative disorders, aneurysms, chordomas, and metastatic lesions (3). Among these, intracranial aneurysms with sellar extension represent a rare clinical entity, accounting for only 1-2% of all intracranial aneurysms (4). The clinical presentation of these vascular lesions is largely determined by their size, anatomical location, and direction of growth.

Unruptured aneurysms typically manifest through mass effect phenomena, most commonly featuring visual pathway compression resulting in visual field deficits such as bitemporal hemianopsia, oculomotor nerve palsy presenting with diplopia and ptosis, trigeminal nerve compression causing facial hypesthesia or neuralgia, and chronic headache. In contrast, ruptured aneurysms present with acute hemorrhagic symptoms including thunderclap headache, nausea, vomiting, and deterioration of consciousness. Endocrinological manifestations, particularly hypopituitarism manifesting as hypogonadism, fatigue, or hyperprolactinemia, occur relatively infrequently and often lead to misdiagnosis as pituitary adenomas.

The current case illustrates an unusual presentation where visual impairment and diabetes insipidus constituted the initial manifestations. The investigations suggested antidiuretic hormone deficiency secondary to compression of the pituitary stalk, posterior pituitary gland, and optic chiasm by a giant internal carotid artery aneurysm (5). This presentation underscores the critical importance of maintaining a high index of suspicion for vascular lesions when evaluating sellar masses, particularly those accompanied by diabetes insipidus. The routine implementation of CTA in preoperative planning is essential to exclude vascular pathologies (6).

Neuroimaging plays a fundamental role in distinguishing sellar aneurysms from neoplastic conditions. MRI typically demonstrates a flow void phenomenon, appearing as a rounded hypointense signal on both T1- and T2-weighted sequences. However, partially thrombosed aneurysms present diagnostic challenges, exhibiting heterogeneous signal characteristics with peripheral enhancement that may mimic solid tumors. Giant aneurysms can produce bony erosion patterns resembling invasive pituitary adenomas. Non-invasive vascular imaging modalities including Magnetic Resonance Angiography(MRA)and Computed Tomography Angiography(CTA) effectively demonstrate the vascular architecture, with contrast-enhanced studies showing simultaneous enhancement of the aneurysm with the parent artery. Three-dimensional reconstructions provide valuable information for surgical planning, while Digital Subtraction Angiography(DSA) remains the diagnostic gold standard, offering both diagnostic clarity and potential therapeutic intervention. Preoperative exclusion of vascular anomalies is an indispensable safety consideration in the management of sellar masses.

The pathophysiological mechanisms underlying diabetes insipidus in such cases are multifactorial. Primary mechanisms include: 1) Mechanical compression of the pituitary stalk disrupting axonal transport of antidiuretic hormone. The pituitary stalk contains numerous axons originating from the supraoptic and paraventricular nuclei, which facilitate hormone transport to the neural terminus. The vascular supply through the superior hypophyseal artery capillary plexus provides essential nourishment and regulatory function. These neural structures demonstrate particular vulnerability to mechanical compression and ischemic injury; 2) Compression of the posterior pituitary gland and its vascular supply compromises the hormone storage and release apparatus. The posterior pituitary serves as the principal reservoir for antidiuretic hormone, consisting of axonal terminals, capillary networks, and specialized pituicytes. Aneurysmal compression causes direct mechanical distortion, structural compromise, and functional impairment of hormone storage and release mechanisms. Concurrent vascular compromise through stenosis or occlusion of the superior hypophyseal artery may induce ischemic necrosis, resulting in rapid tissue deterioration and permanent dysfunction of the antidiuretic hormone system. Ischemic injury represents the most significant factor contributing to permanent, complete diabetes insipidus (7).

Therapeutic management of sellar aneurysms primarily involves endovascular interventions, including coil embolization or flow diversion techniques, and microsurgical approaches such as clip ligation. Isolated flow-diverting stent deployment typically results in gradual aneurysm occlusion over months to years, during which time persistent mass effect may continue to cause visual and endocrinological deficits. Supplemental coil embolization provides immediate aneurysm sac occupation, creating mechanical support that promotes wall retraction, reduces wall tension, and rapidly alleviates compression on adjacent structures. Procedure-related risks include cerebral infarction, thromboembolic events, and access site complications, though experienced centers report major complication rates of 1-5% for elective procedures, depending on aneurysm complexity. The overall prognosis remains favorable, with most patients achieving functional recovery and normal life expectancy following successful treatment of unruptured aneurysms.

Neurological deficits secondary to mass effect depend on the duration and degree of compression. Visual function may show significant recovery following decompression if neural compression has been brief, while prolonged compression resulting in optic atrophy typically yields poor visual recovery (8, 9). Endocrinological dysfunction, particularly diabetes insipidus, presents greater prognostic challenges. While compressive edema may reverse with decompression, ischemic or mechanical disruption of the hypothalamic-pituitary axis often necessitates permanent hormone replacement therapy (10). Current literature describes occasional cases of partial pituitary function recovery following decompression, with rare instances of complete restoration, though the majority of patients experience permanent endocrine dysfunction requiring long-term management. Our patient’s postoperative course has been consistent with these observations, necessitating ongoing hormone replacement therapy.

## Conclusion

Although sellar aneurysms presenting with diabetes insipidus as the initial manifestation remain exceptionally rare, they must be considered in the differential diagnosis of sellar masses. Misdiagnosis as pituitary adenoma with subsequent transsphenoidal or transcranial surgery could lead to catastrophic outcomes. The exclusion of vascular lesions represents an absolute prerequisite before any surgical intervention or biopsy procedure. When conventional MRI reveals atypical features, CTA or DSA must be pursued for definitive diagnosis. The successful management of these complex cases requires dedicated collaboration among professionals from neurology, endocrinology, radiology, and neurointervention disciplines (11).

## Data Availability

The original contributions presented in the study are included in the article/supplementary material. Further inquiries can be directed to the corresponding author.
